# Arbovirus surveillance: first dengue virus detection in local *Aedes albopictus* mosquitoes in Europe, Catalonia, Spain, 2015

**DOI:** 10.2807/1560-7917.ES.2018.23.47.1700837

**Published:** 2018-11-22

**Authors:** Carles Aranda, Miguel J. Martínez, Tomas Montalvo, Roger Eritja, Jessica Navero-Castillejos, Eva Herreros, Eduard Marqués, Raúl Escosa, Irene Corbella, Esther Bigas, Lluís Picart, Mireia Jané, Irene Barrabeig, Núria Torner, Sandra Talavera, Ana Vázquez, Mari Paz Sánchez-Seco, Núria Busquets

**Affiliations:** 1 SCM, Baix Llobregat Council, Sant Feliu de Llobregat, Spain; 2 IRTA, Centre de Recerca en Sanitat Animal (CReSA, IRTA-UAB), Campus de la Universitat Autònoma de Barcelona, Bellaterra, Spain; 3 These authors contributed equally to this work; 4 Department of Microbiology, Hospital Clínic of Barcelona, Universitat de Barcelona, Barcelona, Spain; 5 ISGlobal, Barcelona Centre for International Health Research (CRESIB), Hospital Clinic of Barcelona, Universitat de Barcelona, Barcelona, Spain; 6 Servei de Vigilància i Control de Plagues Urbanes, Agencia de Salud Pública de Barcelona, Barcelona, Spain; 7 CIBER de Epidemiología y Salud Pública, CIBERESP, Instituto de Salud Carlos III, Madrid, Spain; 8 SCM, Badia de Roses i Baix Ter, Empuriabrava, Spain; 9 Consorci de Polítiques Ambientals de les Terres de l’Ebre (COPATE), Amposta, Spain; 10 Secretaria de Salut Pública, Departament de Salut, Generalitat de Catalunya, Barcelona, Spain; 11 Sub-directorate of Surveillance and Response to Public Health Emergencies, Public Health Agency of Catalonia, Barcelona, Spain; 12 National Center for Microbiology, Instituto de Salud Carlos III, Madrid, Spain

**Keywords:** *Ae. albopictus*, transmission, dengue virus, arbovirus surveillance, Europe

## Abstract

Dengue has emerged as the most important viral mosquito-borne disease globally. The current risk of dengue outbreaks in Europe appeared with the introduction of the vector *Aedes albopictus* mosquito in Mediterranean countries. Considering the increasing frequency of dengue epidemics worldwide and the movement of viraemic hosts, it is expected that new autochthonous cases will occur in the future in Europe. Arbovirus surveillance started in Catalonia in 2015 to monitor imported cases and detect possible local arboviral transmission. During 2015, 131 patients with a recent travel history to endemic countries were tested for dengue virus (DENV) and 65 dengue cases were detected. Twenty-eight patients with a febrile illness were viraemic, as demonstrated by a positive real-time RT-PCR test for DENV in serum samples. Entomological investigations around the viraemic cases led to the detection of DENV in a pool of local *Ae. albopictus* captured in the residency of one case. The sequence of the DENV envelope gene detected in the mosquito pool was identical to that detected in the patient. Our results show how entomological surveillance conducted around viraemic travellers can be effective for early detection of DENV in mosquitoes and thus might help to prevent possible autochthonous transmission.

## Introduction

The rapid geographical spread of invasive mosquitoes and vector borne diseases (VBD) as well as their increasing burden are global concerns [1]. Dengue has emerged as the most important viral mosquito-borne disease globally. In a study from 2013, Bhatt et al. [2] estimated that there were 390 million human dengue infections per year worldwide, including 96 million with clinical manifestations. Dengue is caused by dengue virus (DENV), which has four different serotypes (DENV-1 to DENV-4), each of which is further subdivided into distinct genotypes [3]. 

DENV is transmitted through the bites of infected *Aedes* mosquitoes [3] and is present in tropical and subtropical regions of the world. The disease is endemic in more than 100 countries in south-east Asia, the Americas, the western Pacific, Africa and the Eastern Mediterranean. Demographic- and anthropogenic-driven environmental changes combined with globalisation and inefficient public health measures are considered the principal driving forces for the emergence and global spread of dengue in the past 40 years [4].

At the beginning of the last century, dengue had disappeared from Europe [5]. The disease had been present in Spain at least from 1778 and probably until 1927 [5] and the last European outbreak had occurred in Greece in the years 1927–28. During this epidemic, DENV had been transmitted by *Ae. aegypti* and had caused 1 million cases with more than 1,000 deaths [6]. Following the elimination of *Ae. aegypti* in the first half of the 20^th^ century in Europe [7], the risk of dengue mainly re-emerged with the introduction of *Ae. albopictus* in certain areas [8]. Nevertheless, it is noteworthy that on the island of Madeira *Ae. aegypti* presence has been known since 2005 and autochthonous occurrence of dengue was reported there in 2012 [9,10].

*Ae. albopictus* is an Asian mosquito species, which is a vector of several arboviruses including DENV and chikungunya virus (CHIKV). Through the global trade of used tyres and other goods, it began to expand worldwide in the 1970s [11]. From this period, it started to spread to parts of Europe, particularly near the Mediterranean Sea [8]. Travellers, who are infected with DENV or CHIKV in endemic areas of the world, and who return to European areas colonised by *Ae. albopictus*, increase the risk of introduction of the respective diseases, particularly if they are in the viraemic phase. Concerns about such a risk arose with chikungunya outbreaks in Italy in 2007 [12]. 

Subsequently, in the late summer of 2010, an autochthonous dengue case was reported in Nice, France. The likely index case was described as a man who lived in the proximity of the first case, and who had recently returned from Martinique where he contracted DENV infection [13]. Also in August 2010, a 72 year-old man in Germany was diagnosed with DENV infection following his return from a holiday in Croatia [14]. Because shortly after, another DENV infection was detected in the same Croatian village than the one visited by the German tourist, an autochthonous DENV circulation was suspected in that area of Croatia, prompting local investigations. Based on molecular analysis, the virus was probably introduced by a person who arrived in Croatia from the Indian subcontinent in 2010 [15]. Further autochthonous occurrences of dengue in Europe occurred in France in 2013 [16] and in 2015 [17] with probable index cases originating from Guadeloupe and French Polynesia respectively. 

Considering the increasing frequency of dengue epidemics worldwide and the movement of viraemic hosts, it is expected that new autochthonous cases will continue to occur in the future in Europe where the vectors are present. 

In Catalonia (north-eastern Spain), *Ae. albopictus* was first detected in 2004, corresponding to what is considered the first introduction of this species on the Iberian Peninsula and in Spain [18]. Because diagnosed imported cases of arboviral diseases increased in Catalonia from 2009 to 2013 [19], an epidemiological surveillance for DENV, including entomological surveys of confirmed cases, started in 2015, joining the efforts of different public organisations to carry it out. We present the surveillance conducted around viraemic travellers to early detect DENV in mosquitoes in order to avoid and control potential dengue autochthonous outbreaks. 

## Methods

### Epidemiological surveillance

In Spain, DENV is monitored at the national level, in addition Catalonia has a regional epidemiological DENV surveillance. Following national and regional guidelines for arbovirus surveillance, travellers returning from endemic areas with a clinical syndrome compatible with an arboviral infection were screened for DENV and CHIKV in 2015. The arbovirus surveillance, which takes place during the period of *Ae. albopictus* activity, usually lasts from May to November, however, depending on the entomological situation in a given year, it can be expanded. In this study, travellers screened between April and December in Catalonia are included. The clinical and laboratory notification of cases was submitted to the local public health agency, which in turn alerted the mosquito control services.

The regional protocol for surveillance included patients with a febrile syndrome of less than 7 days of duration returning from tropical and subtropical areas. Samples were submitted for testing to the Microbiology Department at Hospital Clinic of Barcelona (centralised laboratory for testing) which reports to the local public health agency any positive RT-PCR or IgM test. During the first week after the onset of symptoms, serum samples were tested for the presence of DENV RNA. In addition, after the day 4 post-symptom onset, serum samples were also tested for the presence of IgM and IgG against DENV. 

Molecular diagnosis for DENV was performed using a specific real time-RT-PCR [20] that discriminates the DENV serotypes. Generic flavivirus RT-PCR [21] followed by sequencing and a dengue NS1-IgM-IgG detection rapid test (SD diagnostics Inc., Seoul, Korea) were eventually used when additional diagnostic evidence was needed. Antibodies against DENV were detected by a commercial ELISA kit (Panbio dengue IgM Capture ELISA and dengue IgG Indirect ELISA). 

The following case definitions were applied in the regional protocol for arbovirus surveillance. A probable dengue case was a patient who had travelled to a dengue endemic area and presented with (i) fever and at least two of the following symptoms: anorexia, a positive Tourniquet test, arthralgia, leukopenia, myalgia, nausea, rash, any warning sign such as abdominal pain, persistent vomiting or mucosal bleeding and/or (ii) had a positive IgM against DENV. A confirmed dengue case was a probable case who has been laboratory confirmed through virus isolation, detection of the viral genome by RT-PCR or by seroconversion in a second sample collected 2–3 weeks after the first sample. For investigation of autochthonous cases, patients with similar compatible symptoms but with no history of travel to tropical and subtropical areas in the last 30 days were considered. 

From September 2015, dengue became a mandatorily notifiable disease in Catalonia. The data used in the present study were retrieved from the records of the centralised laboratory in charge of DENV testing for the arbovirus regional surveillance programme.

### Entomological surveillance

Entomological inspections were carried out by the *Agència de Salut Pública de Barcelona* (ASPB) in the city of Barcelona, by the *Servei de Control de Mosquits del Consell Comarcal del Baix Llobregat* (SCM) in the province of Barcelona, by *Servei de Control de Mosquits Badia de Roses i Baix Ter* in the Girona province and by *Consorci de Polítiques Ambientals de les Terres de l’Ebre* (COPATE) in the Tarragona and Lleida provinces. 

The entomological inspections were performed in a radius of ca 100 m around the home of each diagnosed DENV viraemic case. This distance was selected based on the flight range of *Ae. albopictus* [22]. Inspection of the home address was conducted only with permission of the owner and within 10 days after DENV diagnostic, which was considered the duration of the viraemia. When permission was not obtained, mosquitoes were sampled in the neighbourhood of the residence when possible. Other places such as workplace were inspected if these had been reported to be visited by the case during the viraemic period. 

Entomological monitoring activities were carried out during the activity period of *Ae. albopictus* in the region, which usually starts in May and tapers off in November with activity peaks in July–September, in agreement with the *Ae. albopictus* phenology in other Mediterranean countries [8]. Adult mosquitoes were captured using BG Sentinel traps (Biogents GmbH, Regensburg, Germany) during time intervals of 24 or 48 hours, as well as entomological aspirators (Improved Prokopack Aspirator, Mod. 1419, John W. Hock Company, Florida, United States (US) and CDC Backpack Aspirator Mod. 2846, BioQuip, California (CA), US).

All accessible breeding larval sites were identified and treated if possible using a formulation of *Bacillus thuringiensis israelensis* and *Bacillus sphaericus* (Vectomax FG, Valent Biosciences Corporation, Libertyville, Illinois (Ill), US). Dissemination of information on mosquito prevention and control was conducted in the neighbourhood of viraemic cases’ residences, including instructions on how to eliminate mosquito breeding sites. When high densities of adults were found (continuous presence of biting females during the surveys), adulticide treatments were conducted if they were considered to be effective in terms of mosquito population reduction. 

The female mosquitoes, captured between June and November 2015 in the context of this study, were identified and pooled into groups of up to 35 individuals according to the date and site of collection. The mosquito pools were kept in cell culture media (DMEM supplemented with 6% penicillin/streptomycin) and sent refrigerated to the laboratory at *Centre de Recerca en Sanitat Animal* (CReSA) for DENV detection.

### Dengue virus detection from mosquitoes

All mosquitoes collected were quickly processed for the detection of DENV genome. The mosquito samples were homogenised and the viral RNA was extracted using NucleoSpin Virus kit according to the manufacturer’s instructions (NucleoSpin Virus kit, Macherey-Nagel, Düren, Germany). The mosquito pools were screened for DENV using a real-time TaqMan RT-PCR (RRT-qPCR) which allows detecting any DENV serotype using primers and a probe previously described [23]. Amplification was performed using the AgPath One-Step ID RTPCR kit (Ambion-Applied Biosystems, Foster City, CA, US) following the manufacturer’s instructions in Fast7500 equipment (Life Technologies, Austin, Texas (TX), US). Positive and negative controls for both extraction and amplification were used.

The supernatant from the homogenised mosquito pool that resulted positive was sent to the National Reference Laboratory *Centro Nacional de Microbiología* (*Instituto de Salud Carlos III*, Madrid) for confirmation. The viral RNA was extracted using the QIamp Viral RNA Mini kit according to manufacturer instructions (Qiagen GmbH, Hilden, Germany). Two different RT-PCR assays were used for DENV detection: an in house real-time RT-PCR and a conventional RT-nested-PCR [24].

In order to identify the DENV serotype, genetic material from the positive mosquito pool was partially sequenced using the cFD2 and MAMD primers previously described and specific for a fragment of the non-structural protein 5 (NS5) gene [25]. The amplification product was detected by electrophoresis and purified using the QIAquick PCR Purification Kit (Qiagen, Hilden, Germany). Sequencing reactions were performed with ABI Prism BigDye terminator Cycle Sequencing v.3.1 Ready Reaction (Life Technologies, Austin, TX, US), and analysed using an ABI PRISM model 3730 automated sequencer (Life Technologies, Austin, TX, US). Comparisons with published sequences were performed by searches with the basic local alignment search tool (BLAST) programme at the National Center for Biotechnology Information (NCBI) against the complete GenBank database (http://www.ncbi.nlm.nih.gov/BLAST/) to identify the detected agent.

### Dengue virus characterisation and phylogenetic analysis

For genotyping and phylogenetic analysis, the envelope gene (E gene) of both mosquito and human samples was amplified using the primers described in Table 1. Amplification was performed using a one-step RT-PCR kit (Qiagen GmbH, Hilden, Germany) following the manufacturer’s instructions. A total of 5 µL of viral RNA was added to 45 µL RT-PCR mix. The RT-PCR mix contained 1x OneStep RT-PCR buffer, 400 mM of each dNTP, and 20 pmol of each primer. RT-PCR reactions were carried out using an initial reverse transcription step at 50 °C for 30 min followed by a denaturation (94 °C, 15 min) and 40 cycles including a denaturation (94 °C, 30 s), primer annealing (50 °C, 1 min), and primer extension (72 °C, 2.5 min) step. A final incubation was carried out at 72 °C for 10 min. A second amplification reaction (nested PCR) was seeded with 1 µL of the initial amplification product. Amplification was performed using Go Taq PCR kit (Promega, Madison, Wisconsin, US) following the manufacturer’s instructions and 20 pmol of each primer. Nested-PCR reactions were carried out using an initial denaturation (94 °C, 2 min) and 40 cycles of denaturation (94 °C, 30 s), primer annealing (50 °C, 45 s), and primer extension (72 °C, 2.5 min). A final incubation was carried out at 72 °C for 5 min. A phylogenetic tree was constructed with the common amplified E gene fragment (1,398 nt). The sequences were aligned by MUSCLE, and evolutionary distances were calculated by Tamura–Nei (Tn93 + I) model. Phylogenetic dendrograms were constructed using the maximum likelihood method and bootstrap analysis (1,000 replicates) by the Molecular Evolutionary Genetics Analysis programme (MEGA, Version 7).

**Table 1:** Primers used in RT-nested PCR assays and sequencing

Type of PCR and primers		Sequence	Localisation^a^
RT-PCR	EGENE2-S^b^	5’-CTGAAACATGGATGTCATCAGAAGG-3’	758–782
	RRT2	5’-GCYGARGCYARYTTTGARGGRG-3’	2,534–2,555
Nested	FN2	5'-ATGGCRGCDATYYTGGCDYAY-3'	844–865
	RN2	5'-CGKGARTTCATYCCTATCCATGT-3'	2,326–2,348

^a^ The genome positions are given according to each dengue virus serotype prototype Jamaica N-1409 strain (M20558).

^b^ This primer was described by Domingo et al. [43].

The sequences of the E gene obtained in the study were submitted to GenBank Nt Sequence Database under the following accession numbers: MH253296 and MH253297.

## Results

During 2015, 131 patients were screened for DENV in Catalonia. Sixty-six patients resulted negative by RT-PCR and IgM testing and 65 dengue cases were detected between April and December. Of these, most cases (n = 23) were detected in July. Twenty-eight of these patients were viraemic, as demonstrated by the presence of DENV RNA in serum samples (dengue confirmed cases). In addition, IgM antibodies against DENV were detected in 37 patients, who were classified as probable dengue cases. Viraemic cases presented with a febrile syndrome and had visited at least one of the following countries: Colombia, Costa Rica, Dominican Republic, El Salvador, Honduras, Indonesia, Malaysia, Myanmar/Burma, Philippines, Sri Lanka, Thailand. For the cases with a positive IgM, the clinico-epidemiological data were incomplete.

**Figure 1:**
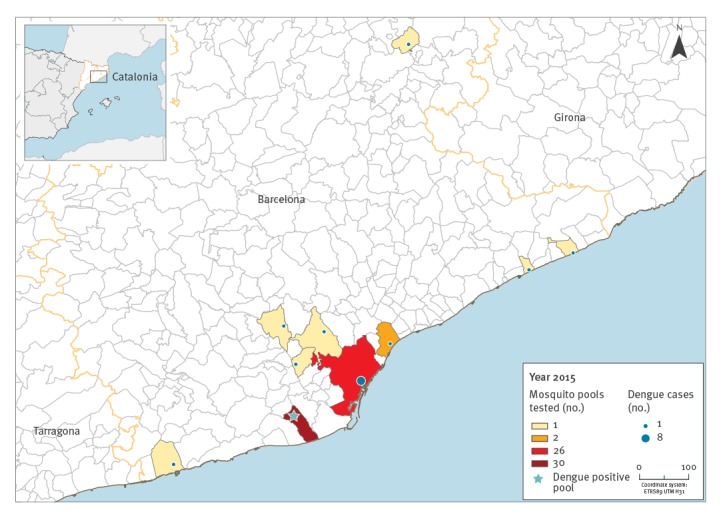
Map of dengue cases (n = 17) around whom mosquito pools (n = 65) were tested for dengue virus, Catalonia, Spain, May–November 2015

Entomological inspections around the DENV viraemic cases were performed in a mean of 12 days after symptom onset (range: 1–18 days) that often started before the arrival to the country and subsequent visit to medical care. During these inspections, female mosquitoes could be trapped in relation to 17 of the 28 cases, either because mosquitoes were present or because access to the mosquito breeding sites was possible (Figure 1). Most breeding sites were abandoned objects with water, including toys, flowerpots or different kinds of scuppers. Six hundred females were obtained from 65 capture sessions performed by BG traps and entomological aspirations. The number of female *Ae. albopictus* mosquitoes per pool ranged from one to 35. The number of mosquito samples per localisation ranged from one to four. 

**Figure 2:**
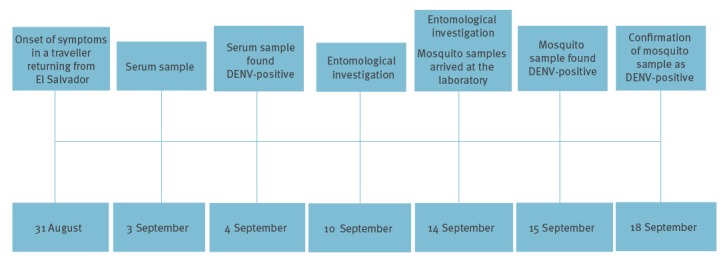
Chronological representation of events leading to dengue virus detection in human and in *Aedes albopictus* samples, Catalonia, Spain, 2015

Of all the mosquitoes collected, only one mosquito pool sampled in the Baix Llobregat region, Barcelona province, on 10 September 2015, was DENV positive (Figures 1 and 2). This pool was retrieved from the residence of a man in his 50s who had travelled to El Salvador. Upon returning from the trip he developed fever, malaise and arthralgia and sought medical attention. On 3 September 2015, serum samples from the patient were submitted to the clinical microbiology laboratory of the Hospital Clinic of Barcelona through the regional surveillance programme for arboviruses in Catalonia. As the samples were collected within 3 days of symptom onset, they were screened for DENV and CHIKV using specific real-time RT-PCR techniques. DENV serotype 2 was detected with a cycle threshold value (Ct) of 16 in serum. On 10 September, an entomological investigation was performed at the patient’s residence, a ground floor house where mosquitoes were abundant. 

DENV: dengue virus.

Two female mosquito pools were collected from the front patio, with 29 and three mosquitoes in each. The first one came from a BG Sentinel trap and the second one from aspiration. The mosquito pool with 29 *Ae. albopictus* females was DENV positive by RRT-qPCR with a Ct of 23.05. The homogenate of the DENV-positive mosquito sample was also confirmed to be positive by two different molecular assays at the laboratory of Arboviruses and Viral Imported Diseases in the *Centro Nacional de Microbiología* (*Instituto de Salud Carlos III*, Madrid). 

**Figure 3:**
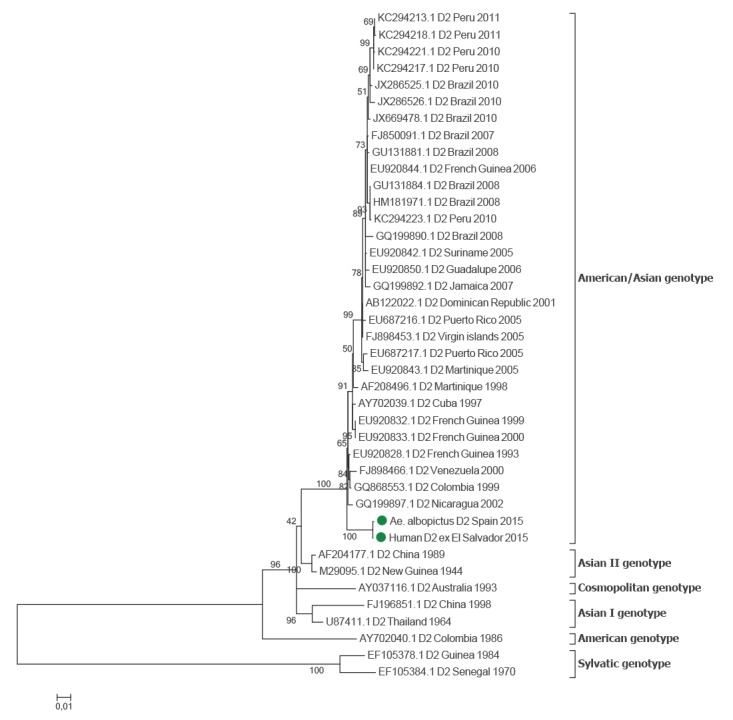
Investigation of the origin of a dengue virus serotype 2 detected in *Aedes albopictus* by phylogenetic analysis of the envelope gene sequence, Catalonia, Spain, September 2015

The DENV detected in the mosquito sample was characterised as DENV serotype 2, as a fragment of its NS5 gene sequence showed a high similarity to those of several DENV serotype 2 isolates from South America. Phylogenetic analysis based on E gene sequences confirmed the identity of the DENV sequences detected in both mosquitoes and patient. The DENV-2 detected was related to other strains of the American/Asia genotype circulating in the Americas (Figure 3).

Strains are denoted by GenBank accession number, place and year of isolation. The green dots indicate the human and mosquito sequences obtained in our study, which were respectively submitted to GenBank under the accession numbers MH253297 and MH253296. The scale bar indicates substitutions per site.

Because a person who lived in the same house as the viraemic patient was diagnosed with multiple chemical hypersensibility syndrome, no chemical treatments were conducted in the house’s patio, although they were carried out in similar patios from contiguous houses. Adults were removed using traps and by aspirations. All possible breeding sites were eliminated. Larvicidal treatments using a formulation of *Bacillus thuringiensis israelensis* and *Bacillus sphaericus* (Vectomax FG, Valent Biosciences Corporation, Libertyville, Ill, US) in all scuppers on the streets in a radius of 100 m around the house of the patient, and adulticide treatments using deltamethrin were performed in contiguous houses’ patio and in an abandoned plot situated in the back of the houses. On the days following the finding of DENV-positive mosquito pools (16, 18 and 21 September) a great effort was made to further sample mosquitoes in the patient’s home and more *Ae. albopictus* (237 females) were collected using BG traps and aspirations, from the same location, all them resulting negative for DENV. Moreover, no autochthonous transmission in humans was reported in this location during the rest of the mosquito activity season in 2015.

## Discussion

The present study shows how epidemiological arbovirus surveillance, including mosquito collection around dengue viraemic cases, can be useful to early detect DENV circulation in local *Ae. albopictus* mosquitoes. The finding of a high viral load of DENV in mosquitoes present at the Baix Llobregat region evidenced that the level of viraemia in the infected traveller from El Salvador was sufficient to efficiently infect local *Ae. albopictus*, highlighting the risk of DENV autochthonous transmission in this area. 

*Ae. albopictus* population density, vector competence, blood feeding behaviour and vector longevity would be determinants for both human-to-mosquito and mosquito-to-human transmission of dengue in Europe. The risk of dengue importation into Europe is greatest in August to October due to a high mosquito activity in that period and a number of passengers coming from dengue-affected areas, some of whom possibly in the viraemic phase [26]. Semenza et al. [26] pointed out that Barcelona, where *Ae. albopictus* is present, is one of the three large European cities with higher risk of dengue transmission. The present study, which finds DENV in *Ae. albopictus* from Barcelona province provides further evidence to this.

Different DENV strains have been involved in autochthonous transmission in Europe since 2010, and all belonged to either serotype 1 or serotype 2 [16,17,27-29]. DENV serotypes 1 and 2 have been the most prevalent DENV serotypes in travellers to Europe during the last years [30]. In the present study, the DENV serotype 2 was characterised as originating from America in both mosquito and human sera samples. 

*Ae. albopictus* can only be unequivocally incriminated as a vector of dengue where transmission occurs in the absence of *Ae. aegypti* or any other potential vector [31]. However, to our knowledge, although *Ae. albopictus* has been implicated as a vector of DENV in all the recent mainland European autochthonous transmissions no DENV detection in local mosquitoes has been reported before our finding. Despite seemingly favourable conditions, places where *Ae. albopictus* predominates over *Ae. aegypti* have never experienced a typical explosive dengue epidemic with severe cases of the disease [32]. Although *Ae. albopictus* is overall more susceptible to DENV mid-gut infection, rates of virus dissemination from the mid-gut to other tissues are significantly lower in *Ae. albopictus* than in *Ae. aegypti*. Therefore, *Ae. albopictus* may play a relatively minor role compared with *Ae. aegypti* in DENV transmission, and this could also be partly due to differences in feeding preferences (i.e. feeding on humans and animals vs humans only) and reduced vector competence [32]. This statement would explain the European autochthonous DENV transmissions in Croatia [27] and France [13,17], as well as those recently detected in Spain in 2018 [33], where *Ae. albopictus* was the suspected vector. Moreover this explanation would also concur with the findings of our study, whereby DENV was detected in *Ae. albopictus* mosquitoes around the imported case in Catalonia, but no authochonous cases occurred. 

Nevertheless, recent examples of rapid arboviral adaptation to alternative mosquito vectors as occurred in the chikungunya outbreak in Indian Ocean islands in 2005 [34] should be taken into account and the possibility of a DENV evolution to be efficiently transmitted by *Ae. albopictus* not disconsidered. Moreover, as already reported for DENV, different vector competence of different mosquito populations from the same mosquito species can exist [35,36]. Therefore, vector competence studies for DENV in different European *Ae. albopictus* mosquito populations such as those reported by Vega-Rua et al. [37], in a mosquito population of France, which showed high efficiency to transmit DENV, will be useful to better estimate the risk of a DENV autochthonous outbreak in a certain area. In fact, a recent study has experimentally shown that an *Ae. albopictus* strain from Catalonia could sustain DENV-2 replication under a simulated Mediterranean temperature regime [38], which would be in agreement with our detection of a high viral load in the DENV-positive mosquito pool in the summer 2015.

Integrated vector management techniques such as source reduction, public education, pesticide application and biological control, produce the optimal control strategy [39], but with limited success if there is a poor participation of communities and a lack of coordination and synchronised implementation [40]. An integrated *Aedes* mosquito control strategy requires the coordinated involvement of local authorities, private partners, organised society and communities. In addition to surveillance of *Ae. albopictus*, maintaining vigilance for any introduction of *Ae. aegypti* species is essential, to assess the risk of mosquito borne diseases and to prepare for the control of disease outbreaks [9].

Early arbovirus detection and effective public health measures reduce the risk of a wider mosquito-borne diseases distribution and increased health impact on the public. Moreover, given the unpredictability of vector-borne disease outbreaks in terms of time, these measures will offer relevant, target-oriented strategies to mitigate problems from their beginning [41]. In case of a dramatic increase of imported viraemic cases, an integrated surveillance could be sustainable if a well-established entomological surveillance framework in coordination with public health authorities was maintained in time. The results of the present study point out that an early intervention is able to detect mosquito infection and to likely reduce the risk of autochthonous transmission as previously recommended by Rezza [42] for areas with relatively cold and dry winters.

The present study highlights the success of an early DENV detection in both human and mosquitoes and the effectiveness of an epidemiological surveillance, which combines epidemiology with ecology, in interrupting local DENV transmission as described by Hubaleck [3]. To sum up, this arbovirus surveillance consisted of different components: (i) routine diagnosis of human disease, (ii) epidemiological investigation, (iii) monitoring and control of *Ae. albopictus* population and (iv) testing mosquito vectors for DENV. However, this surveillance had some limitations: First, complete clinical and epidemiological data were not available for some probable cases and therefore we could not confirm whether they fulfilled all criteria to be classified as dengue cases. On the other hand, the travel history was available for confirmed cases and the data on the viraemic patient and associated DENV-positive mosquitoes were clear. Second, it is possible that asymptomatic dengue cases (imported or autochthonous) may remain undetected. Third, in some instances performing entomological inspections to test mosquitoes for DENV can be difficult due to limited access of the of viraemic patients’ residences .

Arbovirus surveillance relies on multidisciplinarity activities, which are essential for an early detection and prompt implementation of control measures to avoid local arbovirus transmission. Moreover, community engagement is necessary to early detect *Ae. albopictus* in areas where it is not yet established and control its population. Therefore, public health professionals responsible for managing disease outbreaks and policymakers working together with experts from different fields such as virologists, molecular biologists, epidemiologists and entomologists and society as a whole could guarantee a better mosquito-borne disease surveillance. On the other hand, epidemiological surveillances require fluid communication within all the surveillance components and substantial resources that are not always available.

## References

[1] Kraemer MU Sinka ME Duda KA Mylne AQ Shearer FM Barker CM The global distribution of the arbovirus vectors Aedes aegypti and Ae. albopictus. eLife. 2015;4:e08347. 10.7554/eLife.08347 26126267 PMC4493616

[2] Bhatt S Gething PW Brady OJ Messina JP Farlow AW Moyes CL The global distribution and burden of dengue. Nature. 2013;496(7446):504-7. 10.1038/nature12060 23563266 PMC3651993

[3] Guzman MG Harris E Dengue. Lancet. 2015;385(9966):453-65. 10.1016/S0140-6736(14)60572-9 25230594

[4] Ooi EE Gubler DJ Dengue in Southeast Asia: epidemiological characteristics and strategic challenges in disease prevention. Cad Saude Publica. 2009;25(Suppl 1):S115-24. 10.1590/S0102-311X2009001300011 19287856

[5] Angolotti E La fiebre amarilla. Historia y situación actual. La fiebre amarilla en la Barcelona de 1821. [Yellow fever. History and current status. Yellow fever in Barcelona in 1821]. Rev Sanid Hig Publica (Madr). 1980;54(1-2):89-102. 6755634

[6] Reiter P Yellow fever and dengue: a threat to Europe? Euro Surveill. 2010;15(10):19509. 20403310

[7] Rico-Avelló y Rico. Fiebre amarilla en España (Epidemiología histórica). Revista de Sanidad e Higiene Pública XXVII. [Yellow fever in Spain (Epidemiology history). Review of Sanitation and Public Hygiene XXVII]. 1953;(1-2):29-87. Spanish. 24542566

[8] European Centre for Disease Prevention and Control (ECDC). Aedes albopictus. Factsheet for experts. Stockholm: ECDC; 2016. Available from: https://ecdc.europa.eu/en/disease-vectors/facts/mosquito-factsheets/aedes-albopictus

[9] European Centre for Disease Prevention and Control (ECDC). Rapid risk assessment. Update on autochthonous dengue cases in Madeira, Portugal. 20 November 2012. Stockholm: ECDC; 2012. Available from: https://ecdc.europa.eu/sites/portal/files/media/en/publications/Publications/dengue-madeira-risk-assessment-update.pdf

[10] Tomasello D Schlagenhauf P Chikungunya and dengue autochthonous cases in Europe, 2007-2012. Travel Med Infect Dis. 2013;11(5):274-84. 10.1016/j.tmaid.2013.07.006 23962447

[11] Eritja R Escosa R Lucientes J Marquès E Roiz D Ruiz S Worldwide invasion of vector mosquitoes: present European distribution and challenges for Spain. Biol Invasions. 2005;7(1):87-97. 10.1007/s10530-004-9637-6

[12] Rezza G Nicoletti L Angelini R Romi R Finarelli AC Panning M CHIKV study group Infection with chikungunya virus in Italy: an outbreak in a temperate region. Lancet. 2007;370(9602):1840-6. 10.1016/S0140-6736(07)61779-6 18061059

[13] La Ruche G Souarès Y Armengaud A Peloux-Petiot F Delaunay P Desprès P First two autochthonous dengue virus infections in metropolitan France, September 2010. Euro Surveill. 2010;15(39):19676. 20929659

[14] Schmidt-Chanasit J Haditsch M Schoneberg I Gunther S Stark K Frank C Dengue virus infection in a traveller returning from Croatia to Germany. Euro Surveill. 2010;15(40):19677. 10.2807/ese.15.40.19677-en 20946759

[15] Kurolt IC Betica-Radić L Daković-Rode O Franco L Zelená H Tenorio A Molecular characterization of dengue virus 1 from autochthonous dengue fever cases in Croatia. Clin Microbiol Infect. 2013;19(3):E163-5. 10.1111/1469-0691.12104 23279586

[16] Marchand E Prat C Jeannin C Lafont E Bergmann T Flusin O Autochthonous case of dengue in France, October 2013. Euro Surveill. 2013;18(50):20661. 10.2807/1560-7917.ES2013.18.50.20661 24342514

[17] Succo T Leparc-Goffart I Ferré JB Roiz D Broche B Maquart M Autochthonous dengue outbreak in Nîmes, South of France, July to September 2015. Euro Surveill. 2016;21(21):30240. 10.2807/1560-7917.ES.2016.21.21.30240 27254729

[18] Aranda C Eritja R Roiz D First record and establishment of the mosquito Aedes albopictus in Spain. Med Vet Entomol. 2006;20(1):150-2. 10.1111/j.1365-2915.2006.00605.x 16608499

[19] Valerio L Roure S Fernández-Rivas G Ballesteros AL Ruiz J Moreno N Arboviral infections diagnosed in a European area colonized by Aedes albopictus (2009-2013, Catalonia, Spain). Travel Med Infect Dis. 2015;13(5):415-21. 10.1016/j.tmaid.2015.06.008 26169583

[20] Johnson BW Russell BJ Lanciotti RS Serotype-specific detection of dengue viruses in a fourplex real-time reverse transcriptase PCR assay. J Clin Microbiol. 2005;43(10):4977-83. 10.1128/JCM.43.10.4977-4983.2005 16207951 PMC1248506

[21] Moureau G Temmam S Gonzalez JP Charrel RN Grard G de Lamballerie X A real-time RT-PCR method for the universal detection and identification of flaviviruses. Vector Borne Zoonotic Dis. 2007;7(4):467-78. 10.1089/vbz.2007.0206 18020965

[22] Marini F Caputo B Pombi M Tarsitani G della Torre A Study of Aedes albopictus dispersal in Rome, Italy, using sticky traps in mark-release-recapture experiments. Med Vet Entomol. 2010;24(4):361-8. 10.1111/j.1365-2915.2010.00898.x 20666995

[23] Leparc-Goffart I Baragatti M Temmam S Tuiskunen A Moureau G Charrel R Development and validation of real-time one-step reverse transcription-PCR for the detection and typing of dengue viruses. J Clin Virol. 2009;45(1):61-6. 10.1016/j.jcv.2009.02.010 19345140

[24] Domingo C Palacios G Niedrig M Cabrerizo M Jabado O Reyes N A new tool for the diagnosis and molecular surveillance of dengue infections in clinical samples. Dengue Bull. 2004;28:87-95.

[25] Scaramozzino N Crance JM Jouan A DeBriel DA Stoll F Garin D Comparison of flavivirus universal primer pairs and development of a rapid, highly sensitive heminested reverse transcription-PCR assay for detection of flaviviruses targeted to a conserved region of the NS5 gene sequences. J Clin Microbiol. 2001;39(5):1922-7. 10.1128/JCM.39.5.1922-1927.2001 11326014 PMC88049

[26] Semenza JC Sudre B Miniota J Rossi M Hu W Kossowsky D International dispersal of dengue through air travel: importation risk for Europe. PLoS Negl Trop Dis. 2014;8(12):e3278. 10.1371/journal.pntd.0003278 25474491 PMC4256202

[27] Gjenero-Margan I Aleraj B Krajcar D Lesnikar V Klobučar A Pem-Novosel I Autochthonous dengue fever in Croatia, August-September 2010. Euro Surveill. 2011;16(9):19805. 21392489

[28] Wilder-Smith A Quam M Sessions O Rocklov J Liu-Helmersson J Franco L The 2012 dengue outbreak in Madeira: exploring the origins. Euro Surveill. 2014;19(8):20718. 10.2807/1560-7917.ES2014.19.8.20718 24602277

[29] Giron S Rizzi J Leparc Goffart I Septfons A Tine R Cadiou B Nouvelles apparitions de cas autochtones de dengue en région Provence-Alpes-Côte d’Azur, France, août-septembre 2014. [New occurrence of autochthonous cases of dengue fever in Southeast France, August-September 2014]. Bull Epidemiol Hebd (Paris); 2015;(13-14):217-23. Available from: http://www.invs.sante.fr/beh/2015/13-14/2015_13-14_3.html

[30] Neumayr A Muñoz J Schunk M Bottieau E Cramer J Calleri G for TropNet Sentinel surveillance of imported dengue via travellers to Europe 2012 to 2014: TropNet data from the DengueTools Research Initiative. Euro Surveill. 2017;22(1):30433. 10.2807/1560-7917.ES.2017.22.1.30433 28080959 PMC5388098

[31] Gratz NG Critical review of the vector status of Aedes albopictus. Med Vet Entomol. 2004;18(3):215-27. 10.1111/j.0269-283X.2004.00513.x 15347388

[32] Lambrechts L Scott TW Gubler DJ Consequences of the expanding global distribution of Aedes albopictus for dengue virus transmission. PLoS Negl Trop Dis. 2010;4(5):e646. 10.1371/journal.pntd.0000646 20520794 PMC2876112

[33] European Centre for Disease Prevention and Control (ECDC). Rapid risk assessment. Local transmission of dengue fever in France and Spain. Stockholm: ECDC; 2018. Available from: https://ecdc.europa.eu/en/publications-data/rapid-risk-assessment-local-transmission-dengue-fever-france-and-spain

[34] Vazeille M Moutailler S Coudrier D Rousseaux C Khun H Huerre M Two Chikungunya isolates from the outbreak of La Reunion (Indian Ocean) exhibit different patterns of infection in the mosquito, Aedes albopictus. PLoS One. 2007;2(11):e1168. 10.1371/journal.pone.0001168 18000540 PMC2064959

[35] Talbalaghi A Moutailler S Vazeille M Failloux AB Are Aedes albopictus or other mosquito species from northern Italy competent to sustain new arboviral outbreaks? Med Vet Entomol. 2010;24(1):83-7. 10.1111/j.1365-2915.2009.00853.x 20377735

[36] Haddad N Mousson L Vazeille M Chamat S Tayeh J Osta MA Aedes albopictus in Lebanon, a potential risk of arboviruses outbreak. BMC Infect Dis. 2012;12(1):300. 10.1186/1471-2334-12-300 23151056 PMC3519687

[37] Vega-Rua A Zouache K Caro V Diancourt L Delaunay P Grandadam M High efficiency of temperate Aedes albopictus to transmit chikungunya and dengue viruses in the Southeast of France. PLoS One. 2013;8(3):e59716. 10.1371/journal.pone.0059716 23527259 PMC3601061

[38] Brustolin M Santamaria C Napp S Verdún M Rivas R Pujol N Experimental study of the susceptibility of a European Aedes albopictus strain to dengue virus under a simulated Mediterranean temperature regime. Med Vet Entomol. 2018;32(4):393-8. 10.1111/mve.12325 30051490

[39] Abramides GC Roiz D Guitart R Quintana S Guerrero I Giménez N Effectiveness of a multiple intervention strategy for the control of the tiger mosquito (Aedes albopictus) in Spain. Trans R Soc Trop Med Hyg. 2011;105(5):281-8. 10.1016/j.trstmh.2011.01.003 21466887

[40] Baldacchino F Caputo B Chandre F Drago A della Torre A Montarsi F Control methods against invasive Aedes mosquitoes in Europe: a review. Pest Manag Sci. 2015;71(11):1471-85. 10.1002/ps.4044 26037532

[41] Schmidt K Dressel KM Niedrig M Mertens M Schüle SA Groschup MH Public health and vector-borne diseases - a new concept for risk governance. Zoonoses Public Health. 2013;60(8):528-38. 10.1111/zph.12045 23480672

[42] Rezza G Dengue and chikungunya: long-distance spread and outbreaks in naïve areas. Pathog Glob Health. 2014;108(8):349-55. 10.1179/2047773214Y.0000000163 25491436 PMC4394667

[43] Domingo C Niedrig M Gascón J Palacios G Reyes N Malo MJ Molecular surveillance of circulating dengue genotypes through European travelers. J Travel Med. 2011;18(3):183-90. 10.1111/j.1708-8305.2011.00501.x 21539658

